# Multiple receptor tyrosine kinase activation attenuates therapeutic efficacy of the fibroblast growth factor receptor 2 inhibitor AZD4547 in *FGFR2* amplified gastric cancer

**DOI:** 10.18632/oncotarget.2987

**Published:** 2014-12-10

**Authors:** Jinjia Chang, Shanshan Wang, Zhe Zhang, Xinyang Liu, Zheng Wu, Ruixuan Geng, Xiaoxiao Ge, Congqi Dai, Rujiao Liu, Qunling Zhang, Wenhua Li, Jin Li

**Affiliations:** ^1^ Department of Medical Oncology, Fudan University Shanghai Cancer Center, Department of Oncology, Shanghai Medical College, Fudan University, Shanghai, China

**Keywords:** drug resistance, gastric cancer, AZD4547, FGFR2

## Abstract

Fibroblast growth factor receptor 2 (FGFR2)-targeted therapy has attracted considerable attention as novel anticancer agents in gastric cancer (GC). However, intrinsic or acquired drug resistance has emerged as a major challenge to their clinical use. In this study, we demonstrated that several receptor tyrosine kinase (RTK), including EGFR, HER3 and MET, activations contributed to AZD4547 (a selective FGFR2 inhibitor) hyposensitivity in *FGFR2* amplified GC cells. The rescue effect was abrogated by inhibiting these RTKs with their targeted tyrosine kinase inhibitors (TKIs). In addition, synergy in growth inhibition was observed when the GC cells were treated with a combination of AZD4547 and cetuximab (an EGFR monoclonal antibody) both *in vitro* and *in vivo*. More importantly, tissue microarray analysis revealed that these resistance-conferring RTKs were highly expressed in FGFR2 positive GC patients. Taken together, these observations demonstrated RTKs including EGFR, HER3 and MET activations as novel mechanisms of hyposensitivity to AZD4547. It will be clinically valuable to investigate the involvement of RTK-mediated signaling in intrinsicor acquired resistance to FGFR2 TKIs in GC. A combination targeted therapeutic strategy may be recommended for treating *FGFR2* amplified GC patients with these RTK activations.

## INTRODUCTION

Dysregulation of the fibroblast growth factor receptor (FGFR) signaling pathway due to receptor overexpression, gene amplification, mutation or aberrant transcriptional regulation is associated with cancer development and progression in multiple myeloma and cancers of the stomach, breast, bladder, lung, endometrium and prostate [1]. In the case of gastric cancer (GC), FGFR2 protein overexpression by immunohistochemistry [2] was reported to be 30–40%, while the incidence of FGFR2 amplification varies from 3–10% [3-6]. Therefore, FGFR2 amplification may correlate with poor outcomes in patients with diffuse-type gastric cancer [3, 5, 7]. GC cell lines harboring FGFR2 amplification are highly sensitive to FGFR2 inhibitors in preclinical models [4, 8]. Thus, there is significant interest in FGFR2 as a therapeutic target for *FGFR2* amplified GCs, and clinical trials of FGFR inhibitors are ongoing [9].

Several FGFR tyrosine kinase inhibitors (TKIs) such as dovitinib [6], ponatinib [10], NVP-BGJ398 [11] and AZD4547 [8] have been able to inhibit growth of GC cell lines harboring *FGFR2* amplification and shrink tumor xenograft using the same *FGFR2* amplified cell lines implanted in nude mice. Dovitinib is currently being tested in a phase II trial as monotherapy in patients with metastatic or unresectable gastric cancer with either *FGFR2* amplification or polysomy (ClinicalTrials.gov identifier: NCT01719549). The selective FGFR inhibitor AZD4547 is also under a randomized phase II trial comparing AZD4547 to paclitaxel as second line treatment of advanced GC and gastroesophageal junction (GEJ) cancer harboring *FGFR2* amplification or polysomy (SHINE; ClinicalTrials.gov identifier: NCT01457846).

Despite striking preclinical antitumor effects, the long-term efficacy of small molecular TKIs in GC is hampered by the emergence of primary or acquired resistance [12-14]. Previous studies have led to the identification of several TKI resistance mechanisms. One common paradigm is that other RTKs can restore the activation of key intracellular signaling pathways despite inhibition of oncogenic kinase, leading to resistance [15-17]. Recently, we reported that activation of several RTKs were involved in HER2-positive GC unresponsiveness to lapatinib (a HER2 TKI) [14]. However, whether and how other RTK activations cause resistance to FGFR2 inhibitor in GC remains unknown.

In this study, we identified multiple RTK, including EGFR, HER3 and MET, activations as possible mechanisms underlying FGFR2 inhibitor resistance in *FGFR2* amplified GC. We also demonstrated that the combination of AZD4547 (FGFR2 inhibitor) and cetuximab (EGFR monoclonal antibody) offered synergic growth inhibition both *in vitro* and *in vivo*. Our results provide a strong rationale for a combination strategy with agents targeting FGFR2 and other resistance-enriched RTKs.

## RESULTS

### FGFR2-amplified GC cells are selectively sensitive to AZD4547 inhibition

**Figure 1:**
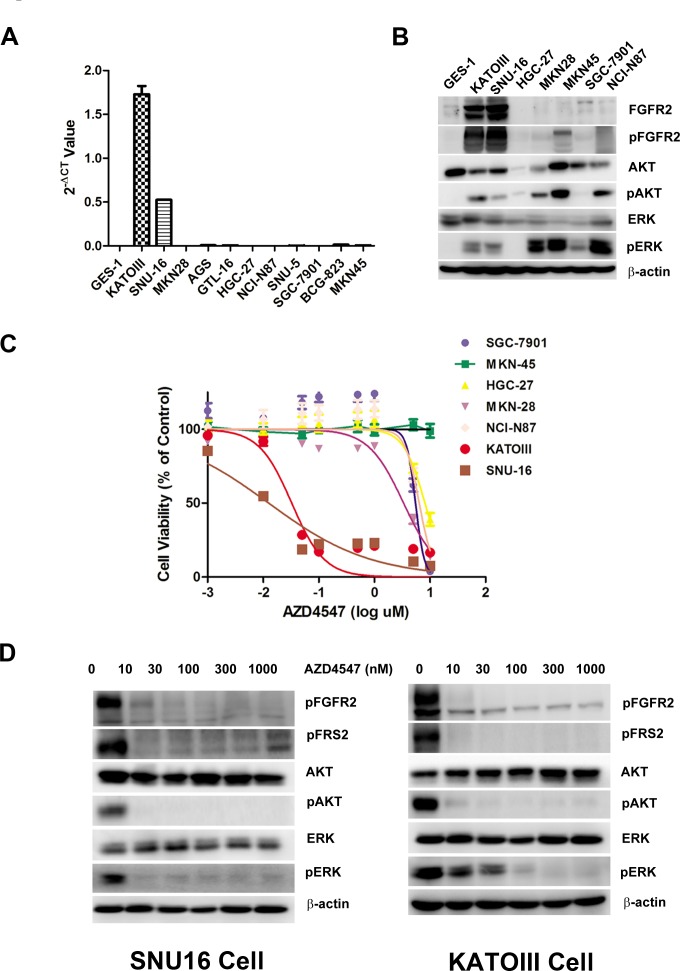
FGFR2 gene amplification predicts AZD4547 sensitivity in GC cells A) Detection of FGFR2 gene amplification in CG cells by qPCR analysis. B) Western blot analyses confirming high expression of FGFR2 proteins from cell lines with FGFR2 gene amplification. C) *FGFR2*-amplified GC cells are selectively sensitive to AZD4547. *In vitro* CCK-8 assay across a panel of 6 GC cells demonstrated that SNU16 and KATOIII cells were extremely sensitive to AZD4547 with IC50 values of 5-10 nM. Data (n = 6) are presented as mean ± SD. D) AZD4547 inhibits FGFR2 pathway activation in SNU16 and KATOIII cells. Cells were incubated with AZD4547 at the indicated doses. Cell lysates were immunoblotted for phospho-FGFR, phospho-FRS2, phospho- and total AKT, and phospho- and total ERK.

To select *FGFR2* amplified GC cells, we first tested a panel of GC cell lines (SNU16, KATOIII, HGC-27, MKN-28, MKN-45, SGC7901 and NCI-N87) for their degrees of *FGFR2* gene amplification and protein expression. As shown in Fig. 1A, quantitative polymerase chain reaction (PCR) determined that SNU16 and KATOIII cells were FGFR2 gene amplified, and the rest of the cell lines were not FGFR2 gene amplified. The degree of *FGFR2* amplification in SNU16 and KATOIII cells corresponded to overexpression of FGFR2 proteins in these cells (Fig. 1B).

To examine the sensitivity of GC cells to a TKI targeting FGFR2, each cell line was exposed to increasing doses of AZD4547 (Fig. 1C). Compared with *FGFR2* non-amplified GC cells, SNU16 and KATOIII cells displayed extreme sensitivity to AZD4547 (Fig. 1C). Fig. 1D shows that a low dose of AZD4547 (10 nM) dephosphorylated FGFR2, FGFR substrate 2 (FRS2), ERK1/2 and AKT in these two cell lines.

### EGFR, HER3 and MET kinase activation attenuates AZD4547 growth inhibition in FGFR2-amplified GC cells

**Figure 2:**
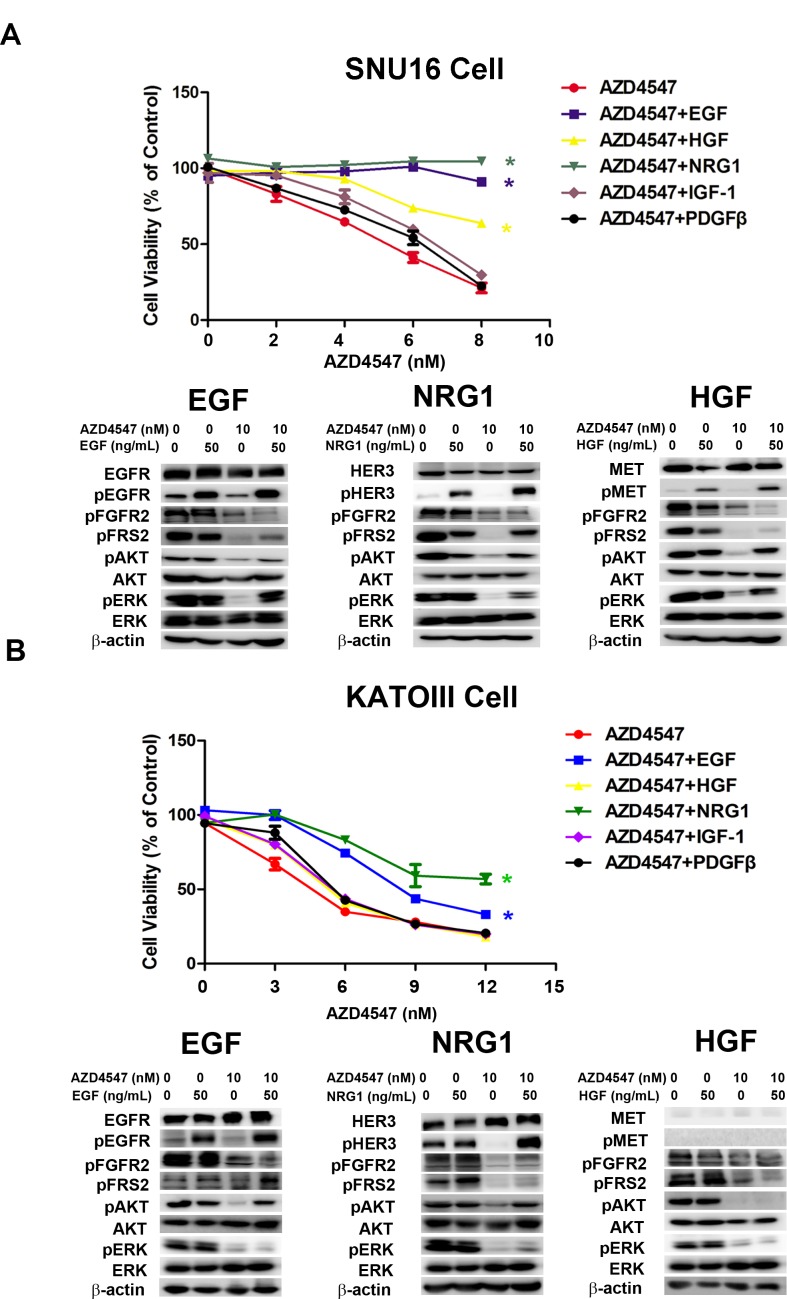
NRG1, EGF and HGF attenuate FGFR2 kinase inhibition in GC cells with FGFR2 amplification A) SNU16 and B) KATOIII cells were treated with increasing doses of AZD4547, either alone or with NRG1, EGF, HGF, IGF-1 and PDGF (50 ng/mL) for 72 hours and then analyzed by CCK-8 assay. Data (n = 3) are presented as mean ± SD. * *p* < 0.01 compared with the AZD4547-treated control group. Cells were treated with 10 nM of AZD4547 for 24 hours, then stimulated with ligands for 30 minutes before whole cell lysates were collected and analyzed by western blot. HER3, MET and EGFR activation attenuated AZD4547 kinase inhibition by restoring MAPK and/or AKT downstream signaling.

To identify RTKs whose activation desensitizes tumor cells to AZD4547, SNU16 and KATOIII cells were treated with AZD4547 (0-10 nM) alone or accompanied by five simultaneous treatments with different ligands, including hepatocyte growth factor (HGF), epidermal growth factor (EGF), platelet-derived growth factor (PDGF), neuregulin 1 (NRG1) and insulin-like growth factor (IGF) (50 ng/mL) for 72 hours. The results showed that NRG1 and EGF rescued both SNU16 and KATOIII cells from AZD4547-induced growth inhibition, whereas HGF abrogated AZD4547 inhibition in SNU16 but not KATOIII cells (Fig. 2A and Fig. 2B). As expected, this ligand-induced AZD4547 hyposensitivity could be blocked by co-targeting the secondary active RTKs (erlotinib: EGFR; AZD8931: pan-HER and PF04217903: MET), confirming that the ligands were acting via their cognate RTKs (Fig. S1).

To investigate the molecular mechanism of RTK ligand-induced AZD4547 hyposensitivity, we examined phosphorylation of FGFR2, EGFR, MET, HER3, FRS2 and the PI3K/AKT and MAPK/ERK pathways by western blotting. As anticipated, AZD4547 inhibited the phosphorylation level of FGFR2, FRS2, AKT and ERK1/2. Importantly, even in the presence of AZD4547, EGF, NRG1 and HGF restored phosphorylation of FRS2, AKT and ERK1/2, respectively (Fig. 2).

Since previous studies indicated heterodimers between RTKs contributed to drug resistance [18], we performed western blot and co-immunoprecipitation analysis in attempt to detect heterodimers between FGFR2 and other certain RTKs. The results revealed that heterodimers between FGFR2 and EGFR/HER3/MET existed in both SNU16 and KATOIII cells. In contrast, SNU16 cells overexpressed MET, whereas MET was barely detectable in KATOIII cells (Fig.S2). Notably, according to the results from CCK-8 assay, HGF could rescue SNU16 not KATOIII cells from AZD4547 treatment. Our results suggested that ligand-induced AZD4547 hypersensitivity is related to basic overexpression of certain RTKs rather than heterodimers distribution.

### EGFR, HER3 and MET activation abrogated AZD4547-induced sub-G1 cell cycle arrest

We then investigated the effect of alternative RTK activations on cell cycle progression of SNU16 cells. Consistent with a previous study [4], AZD4547 inhibited cell growth via sub-G1 cell cycle arrest, as shown by an increased proportion of cells in the sub-G1 phase measured by flow cytometry. The addition of EGF, NRG1 or HGF to AZD4547-treated cells may allow cells exhibiting sub-G1 arrest to enter normal cell cycle progression. This loss of ligand-induced sub-G1 arrest was reversed by secondary RTK inhibitors. We noted the restoration of sub-G1 arrest when cells were treated with AZD4547, RTK ligands and corresponding RTK inhibitors (Fig. S3).

### AZD4547 and cetuximab synergistically inhibited SNU16 cell growth through the MAPK/ERK pathway *in vitro*

**Figure 3:**
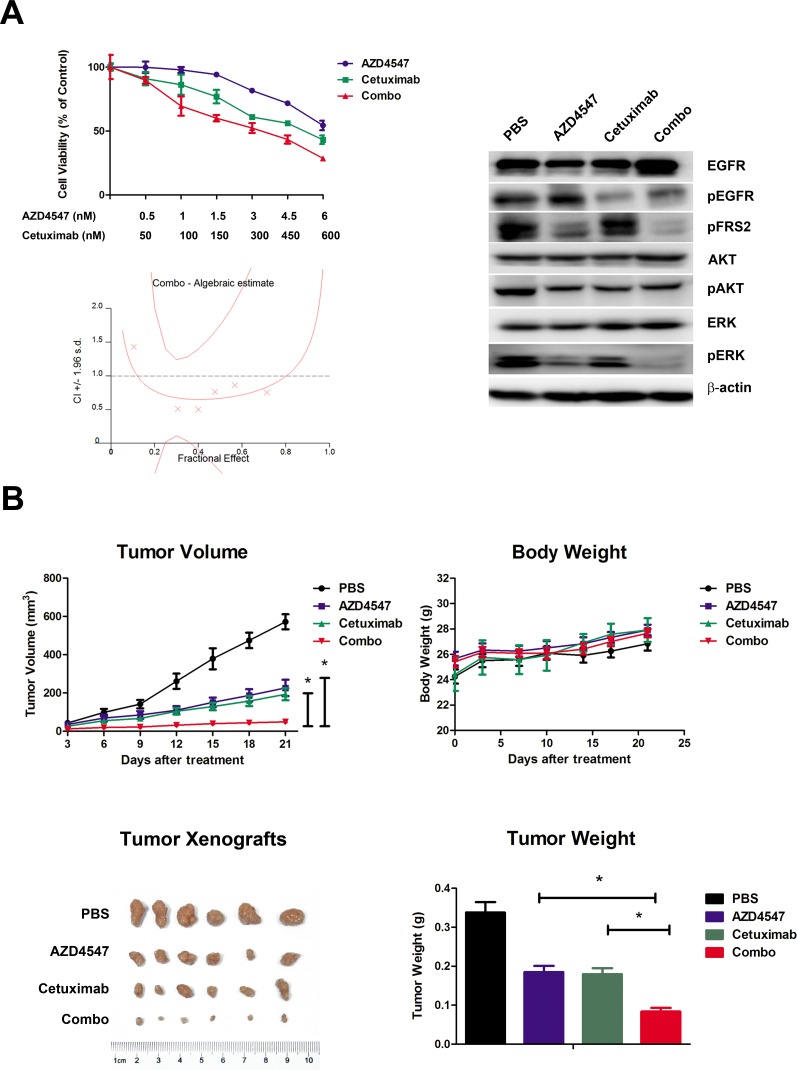
Synergistic antitumor efficacy of AZD4547 and cetuximab on SNU16 cells both *in vitro* and *in vivo* A) Combination therapy with AZD4547 and cetuximab resulted in a significant increase in cell growth toxicity in SNU16 cells. Cells were treated with increasing concentrations of the indicated drugs for 72 hours. The CI/fractional effects curve showed the synergistic anti-proliferative effects of the combination drugs. Multiple drug analyses were conducted by calculating CI values. Data (n = 6) are presented as mean ± SD. * *p* < 0.01 combination therapy vs. AZD4547 or cetuximab alone. SNU16 cells were treated with 10 nM AZD4547 and 1 uM cetuximab for 24 hours, and the whole cell lysates were collected and analyzed by western blot. Combination therapy with AZD4547 and cetuximab synergistically inhibited phosphorylation of ERK1/2. B) Combination therapy with AZD4547 and cetuximab resulted in synergistic antitumor efficacy in SNU16 xenografts. Groups of SNU16 tumor-bearing mice (n = 6) were treated daily with vehicle (PBS), AZD4547 2 mg/kg q.d., cetuximab 1 mg per animal i.v., and AZD4547 2 mg/kg q.d. plus cetuximab 1 mg per animal i.v.. Cetuximab was administrated on days 1, 4, 7, 11 and 14. The tumor volume, tumor weight and animal body weight were measured and calculated as described in the Materials and Methods. Data (n = 6) are presented as mean ± SD. * *p* < 0.001 PBS vs. AZD4547, cetuximab and combination therapy.

To determine whether co-targeting FGFR2 and EGFR exhibits a synergistic effect on SNU16 cells, we examined the effect of individual and combination treatment with AZD4547 (FGFR2 TKI) and cetuximab (EGFR monoclonal antibody) after 72 hours of exposure using the CCK-8 assay. The results showed that the combination of AZD4547 and cetuximab resulted in a significant increase in cell growth toxicity when compared with either drug alone (*p* < 0.01). The combination index (CI)/fractional effect curve showed that the synergistic effects between these two agents became stronger as the concentration increased (Fig. 3A, left).

Next, we analyzed the mechanism of the synergistic interaction between AZD4547 and cetuximab. Western blotting assay showed that administration of AZD4547 or cetuximab alone to SNU16 cells dephosphorylated ERK1/2, whereas their combination resulted in a more profound decrease in phosphorylation of ERK1/2. In contrast, AKT phosphorylation was unchanged by the AZD4547 and cetuximab combination compared with single agent administration (Fig. 3A, right).

### Combined effect of AZD4547 and cetuximab in an SNU16 tumor xenograft model

SNU-16 xenografts were established to evaluate *in vivo* combination effect of AZD4547 and cetuximab. Briefly, animals were treated with a low dose of AZD4547 (2mg/kg, q.d.), cetuximab (1mg per animal, on day 1, 4, 7, 11 14) or AZD4547 plus cetuximab. After treatment for 21 days, AZD4547, cetuximab and their combination significantly decreased tumor volume compared with control group (PBS treated vs. AZD4547, cetuximab or combination treated: 571.5 ± 96.7 mm^3^ vs. 226.6 ± 104.2 mm^3^, 193.18 ± 77.8 mm^3^, 48.9 ± 27.4 mm^3^, respectively; *p* < 0.001). The single agent of AZD4547 or cetuximab gave tumor growth inhibition (TGI) values of 60.4 % and 66.2 %, while their combination resulted in a greater TGI (91.4 %) when compared with AZD4547 or cetuximab alone (*p* < 0.001). Body weight loss related to treatment with AZD4547 or cetuximab was not observed in any of the groups. Taken together, co-treatment of these two drugs demonstrated synergistic activity against SNU16 xenograft without enhanced toxicity (Fig. 3B).

**Figure 4:**
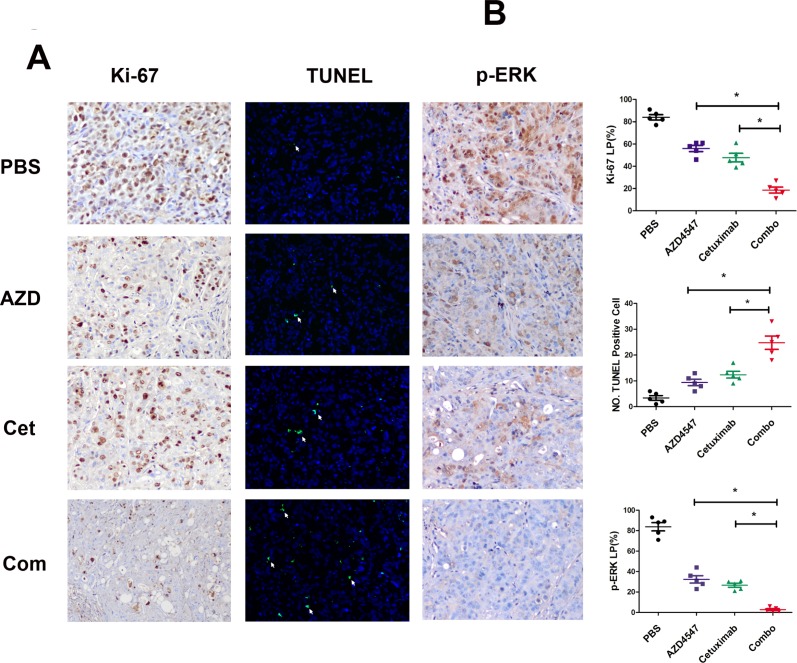
Influence of AZD4547, cetuximab and their combination *in vivo* A) Immunohistochemistry staining of Ki-67 and phosphorylation of ERK1/2 as well as TUNEL assay of SNU16 tumors exposed to various treatments. SNU16 tumors were treated with AZD4547 with or without cetuximab. Note that combination therapy with AZD4547 and cetuximab induced fewer Ki-67 and p-ERK-positive cells (x 200) as well as more TUNEL-positive cells (x 200). AZD, AZD4547; Cet, cetuximab; Com, combination therapy. B) Quantitative analyses of Ki-67 and p-ERK labeling percentage (LP) as well as TUNEL-positive cells are shown. Data (n = 5) are presented as mean ± SD based on three randomly selected high-power microscopic fields for each group. * *p* < 0.05 combination therapy vs. AZD4547, cetuximab or control group.

Next, tumor sections containing viable cells from each treatment group were selected to evaluate the impact of the different therapies on SNU16 cell proliferation and apoptosis (Fig. 4). Scoring of IHC staining for Ki-67 (a nuclear marker for proliferation) demonstrated a statistically significant reduction in proliferation of SNU16 cells in the combination treated vs. PBS, AZD4547 or cetuximab treated groups (18.6 ± 5.9 % vs. 84 ± 5.3 %, 56 ± 6.3 % or 47.8 ± 8.6 %, respectively; *p* < 0.05). Similarly, TUNEL assay staining (marker for apoptosis) analysis revealed that the combined AZD4547 and cetuximab induced a statistically significant increase in SNU16 cells apoptosis (PBS: 3.4 ± 2.1; AZD4547: 9.4 ± 2.7; cetuximab: 12.4 ± 3.0; and combination: 24.8 ± 5.8 TUNEL positive cells, respectively; *p* < 0.05). In addition, the synergistic effect of combinational therapy of AZD4547 and cetuximab *in vivo* might be mediated by downregulating phosphorylation of ERK1/2. As shown in Fig 4, scoring of IHC staining for phosphorylation of ERK1/2 was statistically reduced in the combination treated vs. PBS, AZD4547 or cetuximab treated groups (2.8 ± 2.2% vs. 83.8 ± 9.0 %, 32.4 ± 8.0 % or 26.6 ± 4.4%, respectively; *p* < 0.05).

### EGFR, HER3 and MET are co-expressed in FGFR2 positive GC tumors

**Figure 5:**
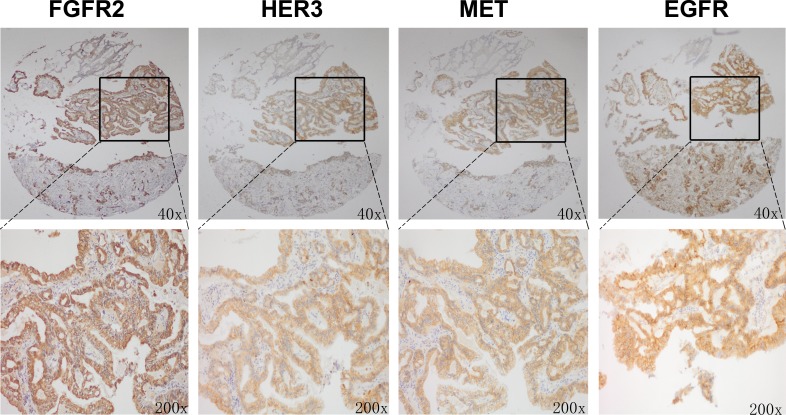
FGFR2 positive tumors showed copositivity of enriched drug resistance related RTKs Representative immunohistochemical analysis of one patient with FGFR2 positive GC is shown. Copositivity of HER3, MET and EGFR were accompanied by FGFR2 positivity.

Our *in vitro* data have demonstrated that FGFR2 TKI resistance is associated with EGFR, HER3 and MET kinase activation. To further validate the clinical significance of our findings, we investigated the expression profile of these RTKs in 145 primary GC surgical samples. Each RTK positivity definition by IHC assay is described in the Materials and Methods. As indicated in Table 1, FGFR2, EGFR, HER3 and MET positivity were detected in 95 (65.5%), 107 (73.8%), 100 (69.0%) and 68 (46.9%) samples, respectively. In the subgroup analysis, in the 95 patients with FGFR2-positive GC, 28 (29.5%), 43 (45.3%) and 16 (16.8%) were EGFR, HER3 and MET positive. In addition, two RTK positives were detected in 16 (16.8%) (EGFR/HER3), 8 (8.4%) (EGFR/MET) and 8 (8.4%) (HER3/MET) cases, respectively. Notably, there were 5 (5.3%) samples that were triple positive (EGFR/HER3/MET), and a typical example with positive staining for each RTK is shown in Fig. 5.

**Table 1:** Distribution of TKRs expression in TMA

Tumor Biomarker Score					
	**HER3**				
**FGFR2**	0	1	2	3	Total (n)
0	15	2	0	0	17
1	11	13	18	1	43
2	22	20	22	4	68
3	5	5	10	7	27
Total (n)	43	40	50	12	145
	**MET**				
**FGFR2**	0	1	2	3	Total (n)
0	17	0	0	0	17
1	18	11	1	3	33
2	32	26	6	4	68
3	12	9	6	0	27
Total (n)	79	46	13	7	145
	**EGFR**				
**FGFR2**	0	1	2	3	Total (n)
0	12	5	0	0	17
1	25	8	0	0	33
2	19	18	20	1	58
3	8	12	5	2	27
Total (n)	64	43	25	3	145

Summary of FGFR2, HER3, EGFR and MET status in gastric cancer tissue microarray (n = 145 primary gastric cancer surgical samples). TKR, tyrosine kinase receptor; TMA, tissue microarray analysis; FGFR2, fibroblast growth factor receptor 2; EGFR, epidermal growth factor receptor; HER3, human epidermal growth factor receptor 3.

**Figure 6:**
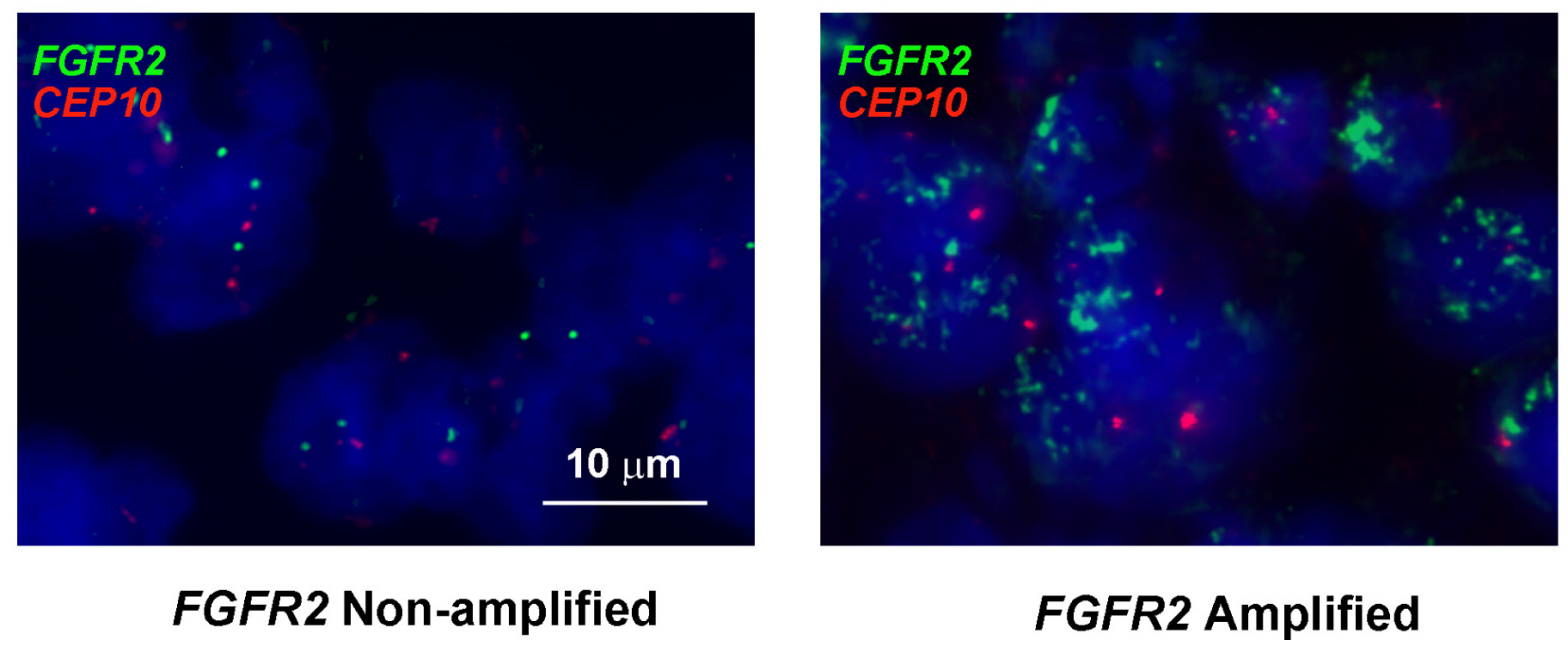
Representative example of FISH analysis of FGFR2 gene status from a cohort of GC specimens (n = 145) Left panel is a non-amplified sample, right is an amplified sample. Red signals represent *CEP10* genes whist green signals are *FGFR2* (original magnification × 1000).

Moreover, we performed FISH analysis to evaluate FGFR2 gene amplification status and its association with FGFR2 as well as other RTKs expression level (see Fig. 6). As a result, 4 samples from a total of 145 samples were identified as *FGFR2* amplified. In these four cases, two cases were FGFR2 2+ while the other two were FGFR2 3+ by IHC assay. Furthermore, the positivity of HER3, EGFR and MET in these four samples were detected in 3 (75%), 3 (75%) and 1 (25%) samples. Taken together, the data from tissue microarray analysis (TMA) in our study revealed that intrinsic resistance-conferring RTKs were commonly enriched in FGFR2 positive GC, indicating the potential clinical application of our preclinical studies.

## DISCUSSION

FGFR2 TKIs such as AZD4547 and BGJ398 are currently under clinical investigation for patients with *FGFR2* amplified GC. Nonetheless, several issues remain, principally for patients who cannot successfully be treated with FGFR2 TKIs due to drug resistance. Due to lack of systematic research, the mechanisms conferring intrinsic resistance to FGFR2 TKIs are poorly defined in *FGFR2* amplified GC.

Recently, we reported that several RTK activations, including MET, HER3, IGF-1R and INSR, conferred lapatinib unresponsiveness in HER2-positive GC [14]. Indeed, “alternative RTK pathway activation” has been considered as a major cause of intrinsic and acquired resistance against several targeted therapies [15, 17, 19, 20]. In the current study, we hypothesized that AZD4547 (FGFR2 TKI) unresponsiveness might also be associated with activation of an alternative RTK pathway and tried to identify the RTKs responsible. To test our hypothesis, five RTKs known to be widely expressed in tumors (EGFR, HER3, MET, PDGFR and IGFR) were assessed. Through cell viability assay, we identified EGFR, HER3 and MET as key pathways modulating unresponsiveness to AZD4547 in *FGFR2*-amplified GC cell lines.

We have herein described a novel mechanism for FGFR2 TKI resistance in *FGFR2* amplified GC through activation of EGFR by its ligand EGF. Specifically, we found that EGF increased growth in the presence of the FGFR2 TKI AZD4547. This EGF-induced AZD4547 hyposensitivity was associated with loss of sub-G1 arrest and phosphorylation of downstream effectors ERK1/2 and AKT. Indeed, FGFR pathway activation by FGFs has been implicated in resistance to EGFR TKIs, including gefitinib and erlotinib, in NSCLC [21]. Thus, it is suggested that crosstalk between FGFR2 and the EGFR pathway mutually affects the efficacy of the single targeted agent. Moreover, dual inhibition of these two targeted therapies might result in a synergistic antitumor effect.

To test our hypothesis, we used AZD4547 (FGFR2 TKI) and cetuximab (EGFR antibody) to block the FGFR2 and EGFR signaling pathways. We showed that dual inhibition of FGFR2 and EGFR activity resulted in a synergistic effect in SNU16 cells through cell viability assay (Fig. 3A). In addition, these *in vitro* observations mirrored the synergistic antitumor activity *in vivo.* The combination of AZD4547 and cetuximab demonstrated striking tumor growth inhibition on SNU16 xenografts (Fig. 3B). This was confirmed by decreased proliferation by Ki-67 staining and increased apoptosis by TUNEL assay (Fig. 4). More importantly, our TMA results showed that 29.5% of FGFR2 positive tumors determined by IHC assay as well as 75% of *FGFR2* amplified tumors identified by FISH analysis were EGFR positive, indicating the potential clinical application of a combination strategy to target both receptors.

Notably, HER3 and MET activation were also found to confer AZD4547 hypersensitivity in *FGFR2-*amplified GC cell lines. These findings echo the results of previous studies demonstrating that NRG1 and HGF were able to rescue KATOIII cells (*FGFR2* amplified) and RT112 cells (*FGFR1* amplified) from another FGFR inhibitor BGJ398 [17]. Furthermore, TMA results showed that in FGFR2 positive tumors, the co-positivity rates of HER3 and MET were 45.3% and 16.8%, respectively. Additionally, FISH analysis showed that in *FGFR2* amplified tumors, 75% and 25% tumors were HER3 and MET positive. Considering the high co-positivity rate of these RTKs in FGFR2 positive GC tumors, our results revealed the possibility of FGFR2 TKI resistance in current and future clinical trials.

An important goal of this work was to show crosstalk between the FGFR2/EGFR, HER3 or MET pathways on 3 levels: firstly, receptors of these pathways form homodimers or heterodimers between each other and lead to transactivation with each other (Fig. S2); secondly, crossphosphorylation of these RTKs leads to reactivated FRS2 phosphorylation, a downstream substrate of the FGFRs (Fig. 2); and thirdly, these RTKs share two common downstream effectors, including ERK and AKT, for which phosphorylation was up-regulated by added ligands (Fig. 2). Notably, variability exists within cell lines harboring *FGFR2* amplification, potentially due to differences in the baseline overexpression of certain RTKs. As shown in Fig. 2 and Fig. S2, compared with SNU16 cells, KATOIII cells presented a relatively lower MET expression level, which could explain why HGF failed to confer drug resistance to AZD4547 in this cell line.

Taken collectively, EGFR, HER3 and MET pathway activation was demonstrated to be related to FGFR2 targeted therapy resistance in *FGFR2* amplified GC. In addition, the TMA results highlighted the clinical implications for GC patients who may acquire resistance to FGFR2 inhibitors. Given that AZD4547 and other potent FGFR inhibitors are now entering clinical practice, our novel approach will be particularly useful in the application of patient selection strategies. More importantly, a synergistic antitumor effect achieved by combining AZD4547 with cetuximab provided a compelling rationale for multi-targeted therapy in *FGFR2* amplified GC.

## MATERIALS AND METHODS

### Cell culture and reagents

NCI-N87, HGC-27 and SGC-7901 human GC cells were obtained from the Cell Bank of Type Culture Collection of Chinese Academy of Sciences (Shanghai, China). KATOIII, SNU16, MKN-45 and MKN-28 GC cells were obtained from 3DBiopharm Biotech Co. Ltd. (Shanghai, China). Cells were cultured in Minimum Essential Medium (HGC-27) or Roswell Park Memorial Institute 1640 medium containing 10% fetal bovine serum (rest of the cells) and 1% penicillin-streptomycin at 37°C in a humidified atmosphere with 5% CO_2_. AZD4547, AZD8931, erlotinib and PF04217903 were purchased from Selleck Chemicals (Houston, TX, USA). Cetuximab was provided by Bristol-Myers Squibb (New York City, NY, USA). Recombinant human HGF, EGF, NRG1, PDGF and IGF-1were purchased from PeproTech (Rocky Hill, NJ, USA). The compounds were dissolved in 100% dimethyl sulfoxide (DMSO; Sigma-Aldrich, St Louis, MO, USA) and diluted with culture medium to the desired concentration, with a final DMSO concentration < 0.2% (v/v). DMSO was also added to the control cells in culture.

### Cell proliferation assay and combination data analysis

Assessment of cell viability was performed as follows. Cells were seeded at 5,000 cells per well in 96-well plates and incubated overnight. Cells were then treated with increasing concentrations of the indicated drugs for 72 hours. Treatments at each concentration were carried out in three replicate wells and repeated three times. Cell viability was determined using the CCK-8 according to the manufacturer's instructions. Half maximal inhibitory concentration was determined by using the non-linear regression model in GraphPad Prism version 5.0 (GraphPad Software, La Jolla, CA, USA).

Combination effects on potency were evaluated using the CI as described previously [22, 23], using Calcusyn version 2.1 (Biosoft, Palo Alto, CA, USA), and derived from the original concept of Chou and Talalay [24]. In general, a CI value < 0.9, 0.9–1.1, or > 1.1 indicates synergy, additivity or antagonism, respectively. CI/fractional effect curves represent the CI versus the fraction of cells affected/killed by drugs in combination.

### Quantitative PCR analysis of FGFR2 genomic amplification

Complementary DNA was prepared from the total RNA of each cultured cell line using QuantiFast SYBR^®^ Green PCR Kit. Real-time (RT)-PCR amplification was carried out using a Mastercycler® pro (Eppendorf, Hamburg, Germany) in accordance with the manufacture's instructions under the following conditions: 95°C for 5 minutes followed by 40 cycles of 95°C for 10 seconds, 55°C for 30 seconds, and 72°C for 1 minute. Primers used for FGFR2 quantitative PCR are listed 5′ to 3′: F-CCCCCTCCACAATCATTCCT, R-ACCGGCGGCCTAGAAAA, and for β-actin: F-CCAGATCATGTTTGAGACCTTC, R-AGGATCTTCATGAGGTAGTCT. β-actin was used to normalize the expression levels in the subsequent quantitative analyses.

### Antibodies and western blotting

Cells were lysed in radioimmunoprecipitation assay buffer supplemented with complete protease inhibitor cocktail (Roche, Basel, Switzerland). Protein concentrations were determined using the BCA protein assay kit (Biyotime, Shanghai, China). Antibodies against p-FGFR2, p-HER3, EGFR, p-EGFR, MET (25H2), p-MET (Tyr1234/1235), p-FRS2 (Tyr436), AKT, p-AKT (Ser473), p42/44 MAP kinase and p-p42/44 MAP kinase (Thr202/Tyr204) were purchased from Cell Signaling Technology (Cambridge, MA, USA). Antibody against HER3 was purchased from Thermo Fisher Scientific (Cheshire, UK). Antibody against β-actin was purchased from Jackson Laboratories (Bar Harbor, ME, USA). Antibody against FGFR2 was purchased from R&D Systems (Minneapolis, MN). Blots were probed with indicated primary antibodies, then incubated with the horseradish peroxidase-conjugated secondary antibody and detected by enhanced chemiluminescence reagent (Pierce, Rockford, IL, USA).

### Immunoprecipitation

Cells were lysed in buffer containing 10 mmol/L Tris (pH 7.5), 100 mmol/L sodium chloride, 1 mmol/L ethylene diamine tetraacetic acid (EDTA), 1% NP40 and protease and phosphatase inhibitor cocktails. FGFR2 was immunoprecipitated from total protein extracts (200 μg) overnight, washed in phosphate buffered saline (PBS) 0.1% Tween 20 buffer, and immunoblotted to detect EGFR, HER3 and MET. Irrelevant immunoglobulin G served as a negative control.

### Cell cycle assay

Propidium iodide (PI) staining was performed to determine the effect of AZD4547 on the cell cycle [25]. Briefly, 3 × 10^5^/mL cells were seeded in 6-well plates and exposed to AZD4547, AZD4547 plus NRG1/HGF/EGF and/or AZD8931/PF04217903/erlotinib for 48 hours. Both floating and trypsinized adherent cells were harvested, fixed in 70% ethanol overnight and resuspended in PBS containing PI (50 mg/mL) supplemented with ribonuclease (100 mg/mL). The DNA content was determined by a Cytomics FC 500 flow cytometer (Beckman Coulter, Miami, FL, USA).

### Nude mice and xenografts

Female, 20-22 g (6-8-week-old) homozygous nude athymic mice were purchased and raised under specific pathogen-free conditions at the Shanghai Laboratory Animal Center, China. All animal work must have been conducted according to relevant national and international guidelines. To develop the human breast xenografts, *in vitro* growing SNU16 cells were harvested by exposure to trypsin-EDTA, washed 3 times with PBS and implanted into the right ﬂanks of the mice (10^6^ cells in 0.1 mL PBS). Once the volume of the tumor xenograft reached approximately 300-500 mm^3^, it was excised and cut into approximately 2 mm^3^ segments, which were further implanted subcutaneously via a Trocar needle into the nude mice [4].

### *In vivo* treatment of AZD4547 and cetuximab

When the SNU16 tumors had reached a mean diameter of around 0.5 cm, usually 8– 10 days after tumor implantation, the mice were assigned to four groups (n = 6 per group): (a) Vehicle; (b) AZD4547 2 mg/kg orally once daily; (c) cetuximab 1 mg per animal intravenously on days 1, 4, 7 and 14; and (d) combination AZD4547 and cetuxizumab. Each animal had its ear marked and was followed individually throughout the experiments. The diameters of the tumors as well as the animal body weights were measured every 3 days, and tumor volume was calculated using the following formula:

V (mm^3^) = V (mm^3^) = π × width (mm) × width (mm) × length (mm)/6

When the designated treatments were completed, the animals were euthanized according to the institutional guidelines. Tumors were resected, weighed and fixed in formalin for paraffin embedding. To evaluate the therapeutic efficacy of the treatment, the tumor growth inhibition rate was calculated using the following equation:

TGI = 100% (mean tumor volume of control group -mean tumor volume of experimental group)/mean tumor volume of control group

Data are representatives of two independent experiments.

### Histology and immunohistochemistry assay

The removed xenograft tumors were fixed with 4% neutral buffered paraformaldehyde and embedded in paraffin before making tissue sections. Patient tissue microarrays were constructed in collaboration with Shanghai Biochip (Shanghai, China), as described previously [26]. Patients' clinicopathologic characteristics are summarized in Table 2. All samples from patients with GC were reviewed histologically after hematoxylin and eosin staining. Representative cores were taken from the paraffin blocks, away from necrotic and hemorrhagic regions. Immunohistochemistry of the paraffin sections was carried out using a 2-step protocol (MaxVision HRP-Polymer Detection System; Maixin, Fuzhou, China) according to the manufacturer's instructions. Briefly, paraffin sections were deparaffinized and then rehydrated. After microwave antigen retrieval, endogenous peroxidase activity was blocked by incubation with 3% hydrogen peroxide. Non-specific binding sites were blocked with PBS containing 10% normal goat serum. The sections were further incubated with the primary antibodies against FGFR2 (R&D systems), MET (Roche), EGFR (Cell Signaling Technology), HER3 (Thermo Fisher Scientific), p-p42/44 MAP kinase (Thr202/Tyr204) (Cell Signaling Technology) and Ki-67 (Dako, Glostrup, Denmark) followed by peroxidase polymer-conjugated secondary antibody. The sections were incubated with diaminobenzidine solution and counterstained with hematoxylin. Negative control slides without the primary antibodies were included for all samples.

**Table 2:** Clinicopathologic characteristics of patients in TMA

Features	No. of Patients	%
Age, years		
Median	59	
Range	29-87	
Gender		
Male	109	75.2
Female	36	24.8
Lauren classification		
Intestinal	95	66.9
Diffuse	47	31.1
Histologic grade		
G1 Well differentiated	1	0.7
G2 Moderately differentiated	56	38.6
G3 Poorly differentiated	84	57.9
G4 Undifferentiated	4	2.8
ATCC TNM stage		
Ⅰ	6	4.1
Ⅱ	37	25.5
Ⅲ	89	61.4
Ⅳ	13	9

Summary of patient clinicopathologic characteristics in gastric cancer tissue microarray (n = 145 primary gastric cancer surgical samples). TMA, tissue microarray analysis.

### Evaluation of immunohistochemical results

Brown staining was evaluated as positive staining. Ki-67 and p-ERK labeling percentages (LP) were evaluated by counting tumor cells in five high-power fields (x 200), and calculated as following: LP = number of Ki-67 labeled nuclei or p-ERK labeled cytoplasm/total number of cells counted x 100%.

TMA immunostaining was scored according to a semi-quantitative 4-grade scale as follows: 0 = no staining or unspecific staining of tumor cells, 1+ = weak (intensity) and incomplete staining (quality) of more than 10% of tumor cells, 2+ = moderate and complete staining of more than 10% of tumor cells, and 3+ = strong and complete staining of more than 10% of tumor cells. All stainings were scored as positive when a clear membrane staining (2+ or 3+) was observed [14, 27, 28]. Slides were independently evaluated by two investigators who were blinded to the clinical information.

### TUNEL assay

TUNEL staining was carried out as described previously [29]. Brieﬂy, 4 μm paraffin-embedded tissue sections were prepared from the human xenograft tumors. The slides were then subjected to TUNEL staining using an in situ cell death detection kit following the manufacturer's protocol (Roche Diagnostics GmbH, Mannheim, Germany). For TUNEL scoring, the average numbers of positive nuclei were calculated in three high-power microscopic fields selected from a central region in a viable tumor, in areas without necrosis.

### Fluorescence in situ hybridisation (FISH) analysis

For FISH analyses, 5 μm sections of the TMAs directly labelled dual-colour DNA probes for *FGFR2* (ZytoVision Ltd., Bremerhaven, Germany) was used. Respective DNA probe sets were applied to the TMA area and incubated overnight at 37°C. Subsequent to several washing steps, nuclei were counterstained with DAPI (4′,6-diamidino-2-phenylindole) and analyzed by epifluorescence microscopy. FISH scoring was performed by counting fluorescence signals in 25 malignant, non-overlapping cell nuclei for each case. The FISH ratio was assessed as the number of genes proportional to the number of centromeres. Amplification of *FGFR2* was defined as a *FGFR2*: chromosome 10 ratio of ≥ 2, or tight FGFR2 gene clusters in ≥ 10% of the nuclei analyzed per tissue section.

### Statistical analysis

Statistical analyses were performed with GraphPad Prism version 5.0 (GraphPad Software, La Jolla, CA, USA). Quantitative variables were analyzed by the Student's t-test or one-way ANOVA with Bonferroni post-test. Differences between cell viability and growth curves were analyzed by two-way ANOVA followed by Bonferroni's multiple comparison test. SD of the mean was indicated for each value by bar. All comparisons with *P* < 0.05 are considered statistically significant.

## SUPPLEMENTARY MATERIAL, FIGURES


