# Supplementary material for: Multiple receptor tyrosine kinase activation attenuates therapeutic efficacy of the fibroblast growth factor receptor 2 inhibitor AZD4547 in FGFR2 amplified gastric cancer
Source: Oncotarget. 2014 Dec 10;6(4):2009–22. doi: 10.18632/oncotarget.2987 (PMC4385832; doi:10.18632/oncotarget.2987)
Supplement: Supplementary file 1 [file oncotarget-06-2009-s001.pdf]

Multiple receptor tyrosine kinase activation attenuates therapeutic efficacy of the fibroblast growth factor receptor 2 inhibitor AZD4547 in *FGFR2* amplified gastric cancer

Supplementary Material

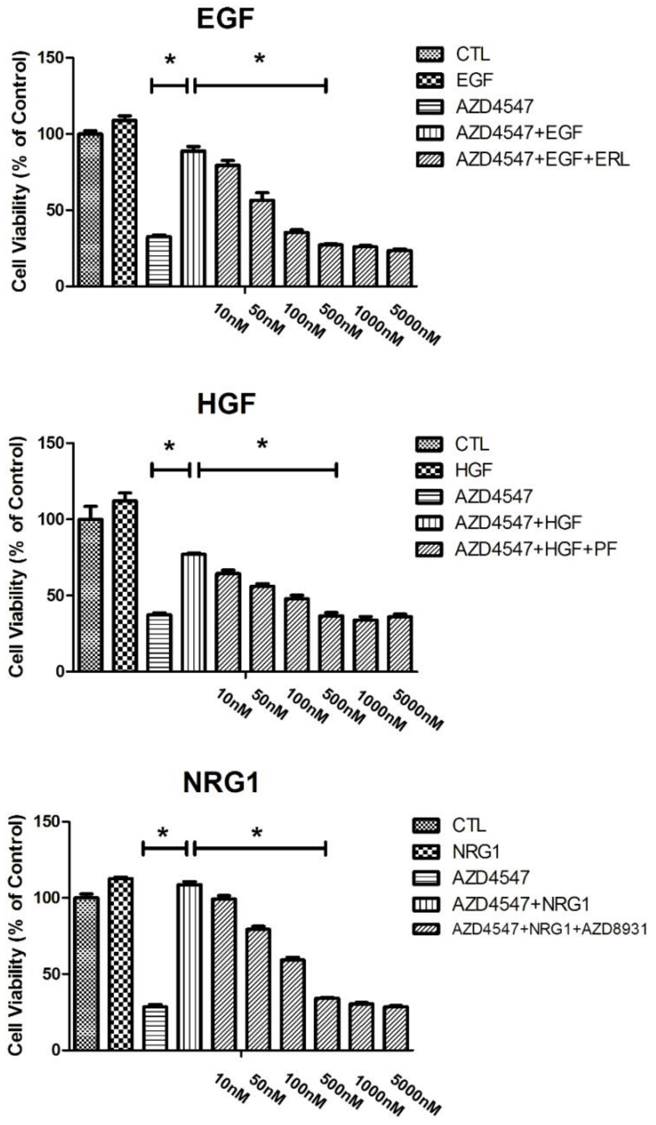

Figure S1: Ligand induced AZD4547 hyposensitivity was reversed by corresponding RTK inhibitors. SNU-16 cells were treated with AZD4547 (5 nM), AZD4547 and ligands (50 ng/mL), or AZD4547, ligands and different concentrations of RTK inhibitor (10 nM-5000 nM) for 72 hours and then analyzed by CCK-8 assay. Note that 500 nM of RTK inhibitors could reverse ligand-induced AZD4547 hyposensitivity. PF, PF04217903; ERL, erlotinib. Data (n = 6) are represented as mean ± SD. *, $p < 0.01$.

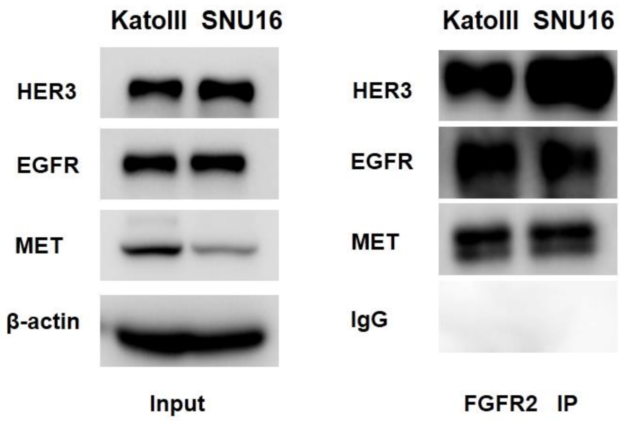

Figure S2: Ligand-induced AZD4547 resistance correlates with baseline overexpression of certain RTK proteins. Left panel: Immunoblots showing the baseline expression status of the indicated RTKs. Right panel: Co-immunoprecipitation showing the distribution of heterodimers between the FGFR2 receptor and indicated RTKs. FGFR2 was immunoprecipitated from whole cell lysates and immunoblotted for the indicated RTKs. Irrelevant IgG serves as a negative control.

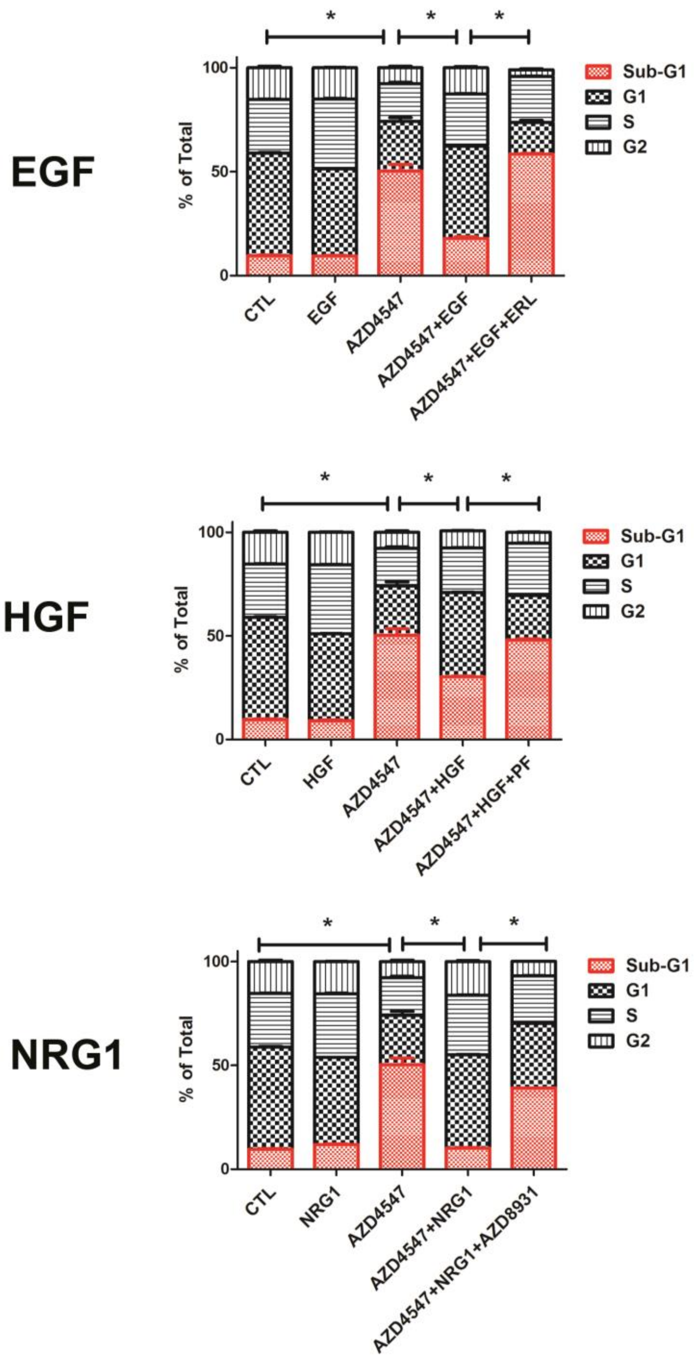

Figure S3: EGF, NRG1 and HGF reversed AZD4547-induced cell cycle arrest in SNU-16 cells. Cells were exposed to AZD4547 (10 nM), AZD4547 and ligands (50 ng/mL) or AZD4547, ligands and second RTK inhibitor (10 μM) for 72 hours and then analyzed for their cell-cycle distribution using flow cytometry. PF, PF04217903; ERL, erlotinib. Data are represented as mean ± SD. *, $p < 0.001$
